# Vehicle detection systems for intelligent driving using deep convolutional neural networks

**DOI:** 10.1007/s44163-023-00062-8

**Published:** 2023-05-02

**Authors:** Rahib Abiyev, Murat Arslan

**Affiliations:** Department of Computer Engineering, Near East University, Mersin-10, North Cyprus Turkey

**Keywords:** Deep learning, Convolutional neural network, Computer vision, Vehicle detection, Intelligent vehicles

## Abstract

In the paper, a vision-based vehicle identification system is proposed for autonomous intelligent car driving. The accurate detection of obstacles (vehicles) during intelligent car driving allows avoiding crashes, preventing accidents, saving people’s lives and reducing harm. The vehicle detection system, which uses low-quality images captured by a monocular video camera mounted at the front of the car, is based on convolutional neural networks (CNN). The CNN can extract global features of the images using convolutional layers and achieves more accurate, and faithful contours of vehicles. The CNN structure proposed in the paper provides high-accuracy detection of vehicle images. The experiments that have been performed using GTI dataset demonstrate that the CNN-based vehicle detection system achieves very accurate results and is more robust to different variations of images.

## Introduction

The rapid growth in the number of vehicles causes us to pay more attention to the traffic safety problem. In the traffic, more accidents may accuer due to lack of awareness or fatigue of drivers. Therefore, the development of a driver assistance system that warns the driver of any dangers on the road and helps to prevent accidents is important. Using computer and vision technologies to detect obstacles, like other cars in front of own car and to make a decision about the turning angle of the car and to warn the driver are the basic functions of these systems. The intelligent car driving system needs the construction of robust vision-based driver assistance and vehicle detection systems. The driver assistance module uses the road map to capture an image of the road. This module needs a video camera for capturing the vehicle images, and a high-performance computer for processing the captured images. The basic aim of this module is to track the road. Vehicle (or obstacle) detection module is widely used in recognizing vehicles in videos or photos during driving cars in traffic. The vehicle detection module uses image processing techniques to analyze the video images, and then isolates and detects these vehicles and tracks them in video frames. The accurate recognition of vehicles is very important for intelligent car driving and depends on traffic volume, lighting conditions, road conditions etc. Segmentation of road surface, recognition of track and then detection, isolation and tracking of the vehicles should be performed in a short time for autonomous driving of the car. These systems will allow decreasing the number of accidents in traffic and improve traffic safety.

A number of research studies have been implemented for improving the performance of vision-based driver assistance and vehicle detection systems. The used methods are very challenging. Optical sensor-based models have been used for the detection of road trucks and vehicles. Optical sensors which are very expensive and have low resolutions can measure at a certain distance, and provide limited data for future processing. Otherwise, video cameras can be used for this purpose. The used cameras can obtain all visual information for a better solution and they are very cheap. The research papers [1–3] use visual information for pedestrian detection [1, 2], lane detection [3], and vehicle detection [4]. Common methods used in vision-based vehicle detection are divided into two steps: (A) Hypothesis Generation (HG) which is to find possible vehicle locations in an image quickly for analysis and (B) Hypothesis Verification (HV) is to verify potential vehicles in an image.

The real-time vehicle detection systems that use video cameras have some challenging threads. While driving the car, lighting conditions can be changed frequently, weather can be unpredictable and vehicles can be in different shapes and colours that lead to reflections. Some vehicle detection methods have been presented using computer vision. MAO et al. [5] developed a technique for vehicle detection using a support vector machine (SVM) and Histogram of Oriented Gradient (HOG). Liang et al. [6] considered mixed Haar Wave Features and HOG with Generalized Multiple Kernel Learning (GMKL) [7]. Experimental results have shown that the combined HOG and Haar descriptor by using GKML is better than when they are used alone without tuning any parameters. Later, some different vehicle detection techniques, such as Gabor filters and SVM were proposed. Zhang et al. [8] developed a system that uses Gabor filters for vehicle feature extraction and SVM for vehicle detection. This system can discard irrelevant details and improve robustness. The paper gives a comparison of the systems based on Neural Network and SVM techniques. It was shown that SVM-based vehicle detection using Gabor filters is faster, and the performance of the system can be increased with the “non-vehicle” class than the “vehicle” class. To this end, the approach requires large datasets. Background subtraction technique using the Gaussian mixture is utilized for object detection [9]. This technique requires many frames to improve performance. The considered methods require more data sets for improving the performances that need more operational time and are not suitable for some tasks. The paper [10] uses the gradient technique and Adaboost classification to design a real-time vision-based vehicle detection system. Toulminet et al. [11] presented a technique for detection and distance computation of preceding vehicles by using stereo-based 3-D feature extraction. Ha et al. [12] proposed traffic parameter extraction method and neural edge-based vehicle detection. The method is effective for vehicle detection and independent of environmental conditions. Zhou et al. [13] used adaptive background estimation and SVMs to classify vehicles. The obtained experimental results indicate the robustness of the method in various conditions. The paper [14] designed a vehicle detection system from static images using colour and edges with a cascade multichannel classifier. The obtained results proved that global colour features and local edge features are well performed for vehicle detection. The reference [15] used visual and radar data and presented a vehicle detection system. The research uses the vertical symmetry technique to search for possible vehicles in search areas. The paper [16] used a hidden Markov model (HMM) to separate the vehicles from their background and to deal with changes in the environment. A robust and effective real-time vehicle detection system is presented. The paper [17] used a combination of colour, edge and motion information and designed a real-time multiple-vehicle detection system. This system uses a recursive least-squares filter to recognize tracks road boundaries and lane markings. The used methods require more data sets for improving the performances that need more operational time and are not suitable for some tasks.

In this study, we proposed a deep CNN for vision-based vehicle detection. Feature extraction, CNN and sliding window method are utilized for detecting vehicles. Le Cun et al. proposed CNN structure [18] for the classification of hand-written numbers from 32 to 32 pixel images. It is called LeNet-5 and it has seven convolutional layers [18, 19]. Afterwards, similar to LeNet, other deep learning structures were designed [21–24]. These are AlexNet [20], ZFNet [21], VGGNet [22], GoogleNet [23] and ResNet [24]. Different CNN-based systems are constructed to solve pattern recognition, image processing, classification, etc. problems. Recently, CNN was utilized for face recognition [25], hand gesture recognition [26], brain-tumor segmentation [27], face sketch synthesis [28], microaneurysm detection [29], fingerprint enhancement [30], segmentation of glioma tumors in the brain [31], handwritten recognition [32], sign language translation[33], segmenting neuroanatomy [34], change detection using heterogeneous optical and radar images [35], prediction of eye fixations [36], improving acoustic source localization [37], short-term wind speed forecasting [38], and showed good results. CNN is applied to solve COVID-19 and pneumonia diagnosis using X-ray images [39], image and video recognition [40], recognition of head movement [41] and vehicle detection [42] problems.

In the paper, a vision-based vehicle detection system is designed. The contributions of the paper include: Using CNN a vision-based vehicle detection system was proposed for autonomous intelligent car driving; The architecture of CNN is designed for a vision-based vehicle detection system; The four CNN models are designed and their performances were analyzed to detect cars at various distances in the traffic; The simulations have been done to demostrate the effictiveness of using the proposed system. The CNN-based vehicle detection developed for intelligent car driving allows to avoid crashes, prevent accidents, save people's lives and reduce damages. The use of vehicle detection and recognition system will enable safe driving and improvement of traffic quality.

The paper is organized as follows. Section 2 describes Convolutional Neural Networks used for vehicle detection. The CNN structure used for vehicle detection is proposed. Section 3 presents the obtained simulation results. Finally, Sect. 4 gives the conclusions.

## Convolutional neural network

**Fig.1:**
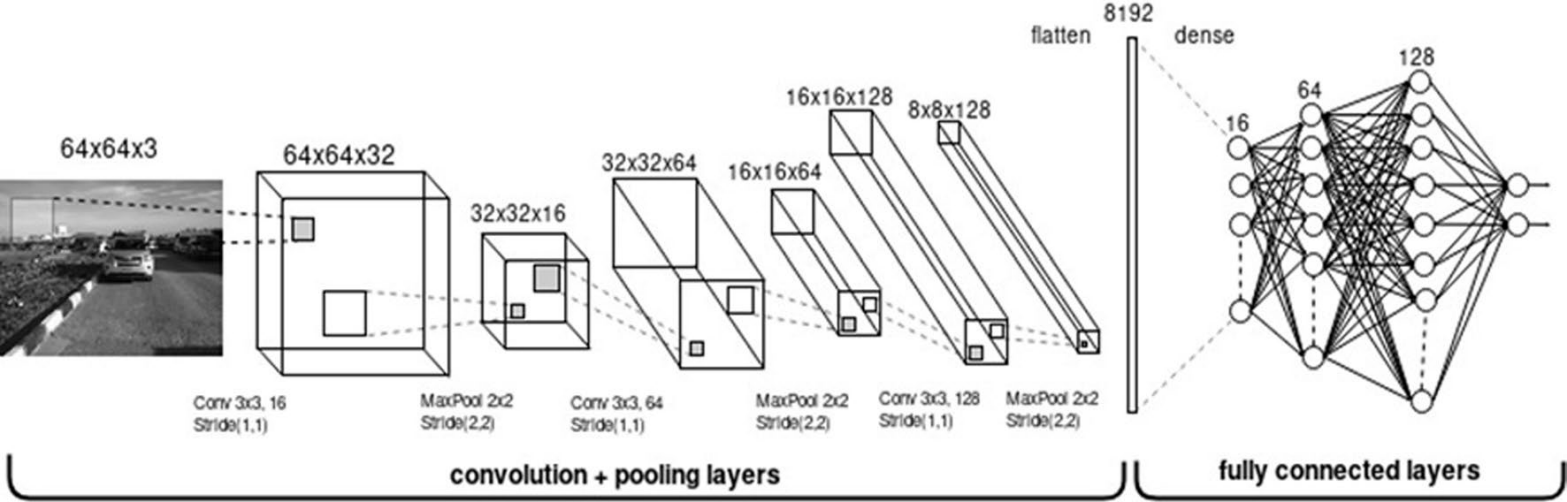
The structure of CNN

CNN includes several hidden layers called convolution, pooling and feedforward and full-connected layers. The convolutional layers that are the main building modules of CNN are used to extract features from the input images. Figure 1 presents a simple CNN structure. The input of the first convolutional layer is an image and its output is the feature space. Next convolutional layer uses this feature map to drive the output feature map. More detailed features of the input images can be extracted by increasing the number of convolutional layers. In the next stage the RelU activation is applied to the ouput feature map. The output features obtained are entered into the pooling layers. Pooling layers are usually used between convolutional layers. The pooling layer reduces the dimension of feature maps. The output features are flattened and converted into a one-dimensional array. These signals are inputs for a fully connected network that perform the classification of input signals.

**Fig. 2:**
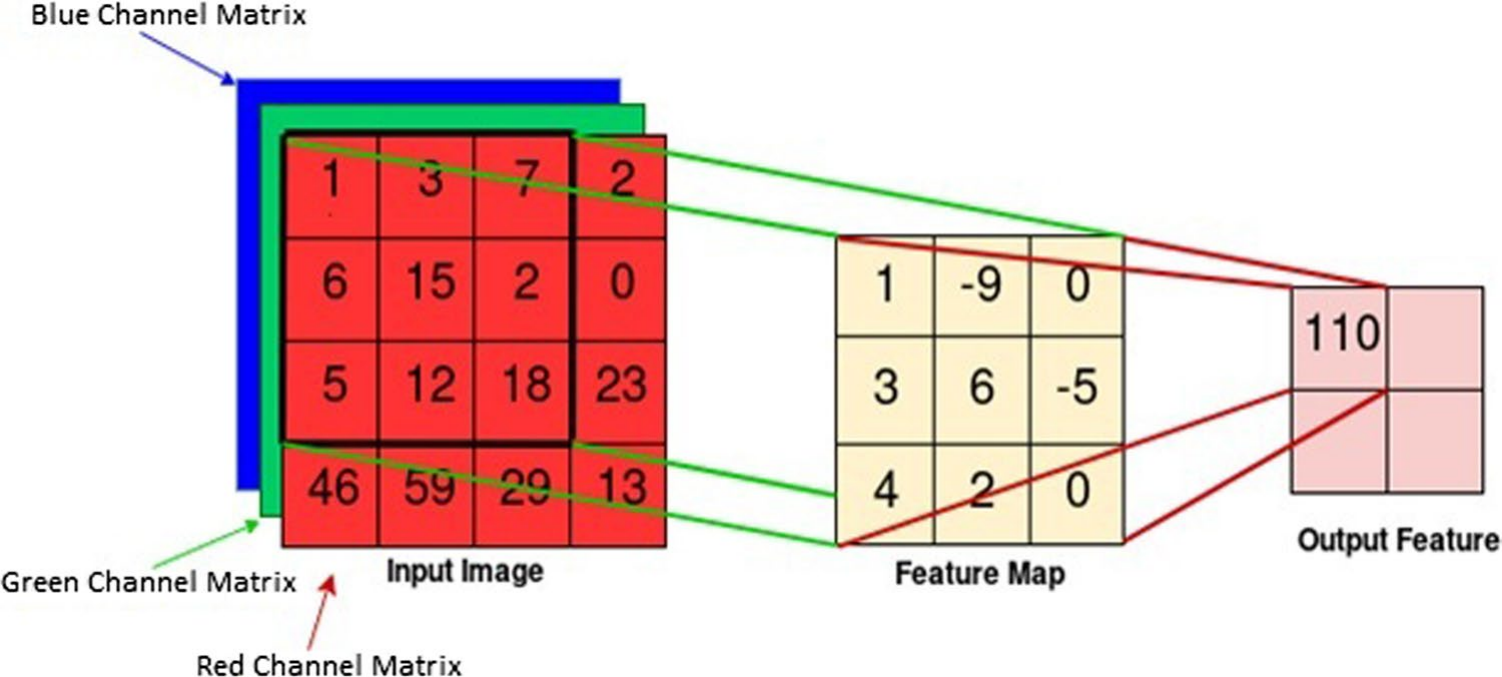
Fragment of convolutional layer

Let’s consider feature extraction and classification stages in detail. The image of size *mxmxq* is the input for CNN*.* Here *m* is the size of the image, *q* is the number of channels, and for RGB image *q* = *3.* The convolutional layer includes k filters (or kernels) of size *pxpxq.* Here *p* should be smaller than *m*. The filters produce *k* feature maps each one having a size of *m-p* + *1*. Each map is pooled with the *min of max* over *pxp* contiguous regions. The value of *p* is usually in the interval [2, 5]. According to the kernel size, the part of the image is selected and the convolution operation is performed. According to the kernel size (assume that *p* = *3*) red matrix is separated into smoller matrices of size 3 × 3 (see Fig. 2). For example using 4 × 4 red matrix and 3 × 3 kernel, for the first 3 × 3 red matrix the following result will be obtained (Fig. 2). Using kernels, the product and then the summation operations will be done. According to the size of the kernel, four matrices of size 3 × 3 will be obtained from the red input matrix. Cell-by-cell product operations will be done between the obtained matrices and kernel (yellow matrix in Fig. 2). As a result of product operation, the four feature matrices will be derived from the red input matrix. For each feature matrix, the sum of cells’ values will be determined. After getting the sum for each matrix, the resulting matrix (last pink matrix) having four cells will be obtained. The same operation will be applied to green and blue matrices also.

**Fig.3:**
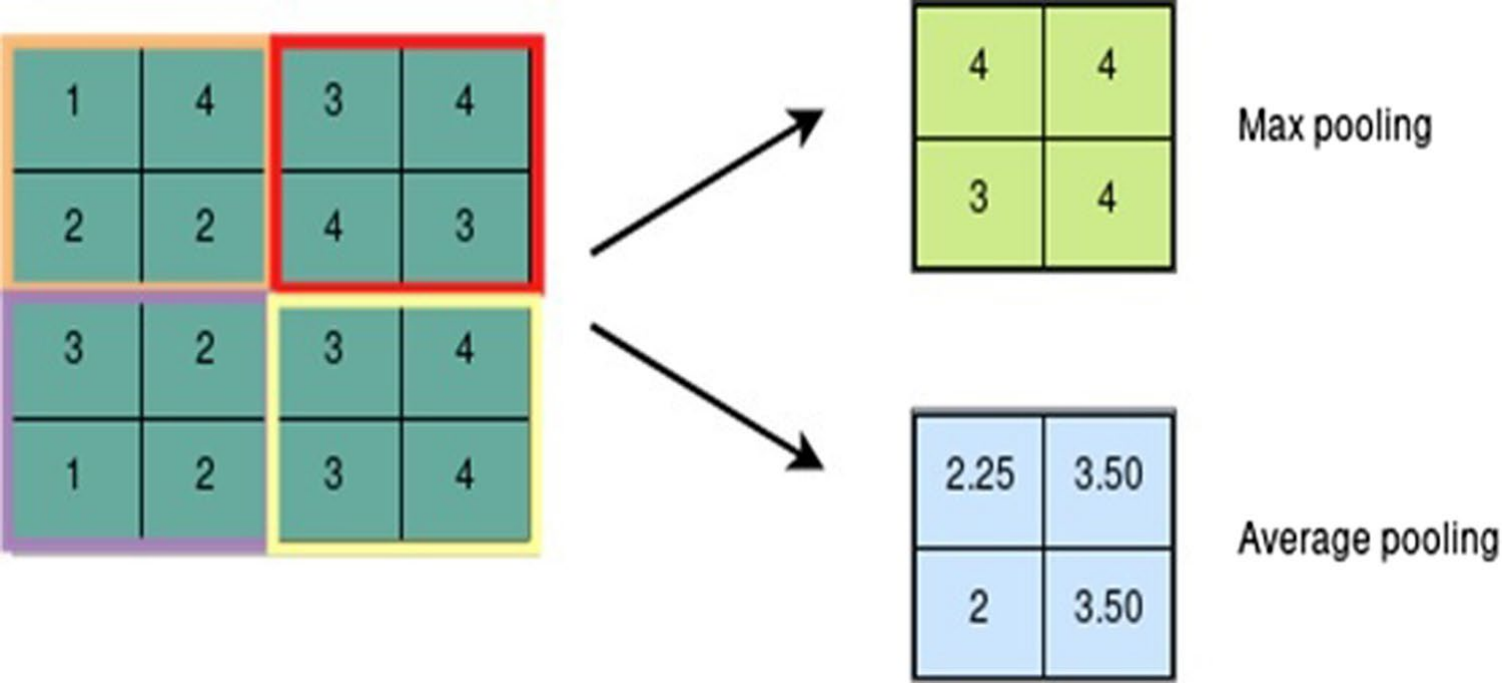
Pooling operations

The resulting matrices will be 2 × 2×3 in size. After these operations, ReLU activation is applied for the nonlinear transformation of input signals. Then *max* (or average) pooling operation is applied to these results. The down-sampling operation is utilized to decrease the size of the feature matrix. As shown in Fig. 3, the 4 × 4 matrix is separated into smaller matrices of size 2 × 2. In the paper, the pooling is carried out using *max* operation. Here for each separated 2 × 2 image, the maximum value is determined. As shown, on the matrix we have [3, 4, 4, 4] values. From these values, 4 is obtained as the maximum value. The results of the pooling layer will be flattened in order to receive the feature vector of the input image. In the figure for max pooling, the flatted vector will be. The input of the fully-connected network is the feature map, and the output will be the clusters.

Let’s consider the mathematical formulation of the operations presented above. The output of the convolutional layer is determined using matrices characterizing the input image and local kernels (filters). The convolutional layers use filters (kernels) to determine the feature map. The *l*-th layer feature value ${z}_{i,j,k}^{l}$ at location (*i,j*) in the *k* th feature map is determined as

1 $${z}_{{\varvec{i}},{\varvec{j}},{\varvec{k}}}^{{\varvec{l}}}={{\varvec{w}}}_{k}^{{l}^{T}}\cdot {{\varvec{x}}}_{i,j}^{l}+{b}_{k}^{l}$$

where ${{\varvec{x}}}_{i,j}^{l}$ are inputs, ${{\varvec{w}}}_{k}^{l}$ are the weights and ${b}_{k}^{l}$ are the bias terms of the *k*th filter of the *l*th layer. The kernel ${{\varvec{w}}}_{k}^{l}$ generates the feature map ${z}_{i,j,k}^{l}$ [44, 45]. Then the non-saturated Rectified Linear Unit (ReLU) activation function is used for the transformation of feature signals.

2 $$a_{i,j,k}^{l} = \,a(z_{(i,j,k)}^{l} )$$

The ReLU activation function prunes the negative values to zero and holds the positive values. The use of the ReLU activation function causes faster calculations and faster results compared to the sigmoid or tanh activation function. Some research works have shown that ReLU can work better than the sigmoid or tanh activation function [43].

In the pooling layer for each feature map ${a}_{:,:,k}^{l}$ the output is determined as:

3 $${y}_{i,j,k}^{l}=pool({a}_{m,n,k}^{l}),\forall (m,n)\in {R}_{i,j}$$

where ${R}_{i,j}$ is a local neighbourhood around the position *(i,j)*. In the paper, we used max pooling for implementation pooling operations.

The last layer of CNN is the Dense layer or fully-connected layer, which implements the classification. The inputs of this layer are individual features presented as flatten layer. This data was converted into a 1D feature vector and fed to fully-connected network for classification purposes. In fully-connected network, the softmax activation function is used. The output signal of the fully-connected network is calculated as.

4 $$y_{i}^{(l)} = f(z_{i}^{(l)} )\,{\text{with}}\,z_{i}^{(l)} = \sum\limits_{i = 1}^{{m_{i}^{(l - 1)} }} {w_{i,j}^{(l)} y_{i}^{(l - 1)} } \,$$

where $f$ is a nonlinear function, $w_{i,j}^{(l)}$ are the network weight coefficients.

After determining CNN’s output signals the learning of *θ* network parameters start. The learning is performed using a loss function calculated at the output of CNN. Using input–output training pairs $\left\{\left({{\varvec{x}}}^{\left(i\right)},{{\varvec{y}}}^{\left(i\right)}\right); i\in \left[1,...,N\right]\right\}$ the learning of θ parameters is carried out in order to minimize the value of the loss function. The loss function is formulated as

5 $$L=\frac{1}{N}{\sum }_{i=1}^{N}l({\varvec{\theta}};{{\varvec{y}}}^{\left(i\right)}{, {\varvec{o}}}^{(i)})$$

where o^i^ and y^i^ are current- and target output signals, correspondingly. Using loss function the *θ* unknown parameters are determined. For this purpose, in the paper, an adaptive moment estimation algorithm, called Adam optimizer is used [44]. For the efficient training of CNN, a large volume of training pairs is required. In the paper, data augmentation is applied for solving overfitting and data scarcity problems and for increasing the performance of CNN.

## Simulation of the system

**Fig. 4:**
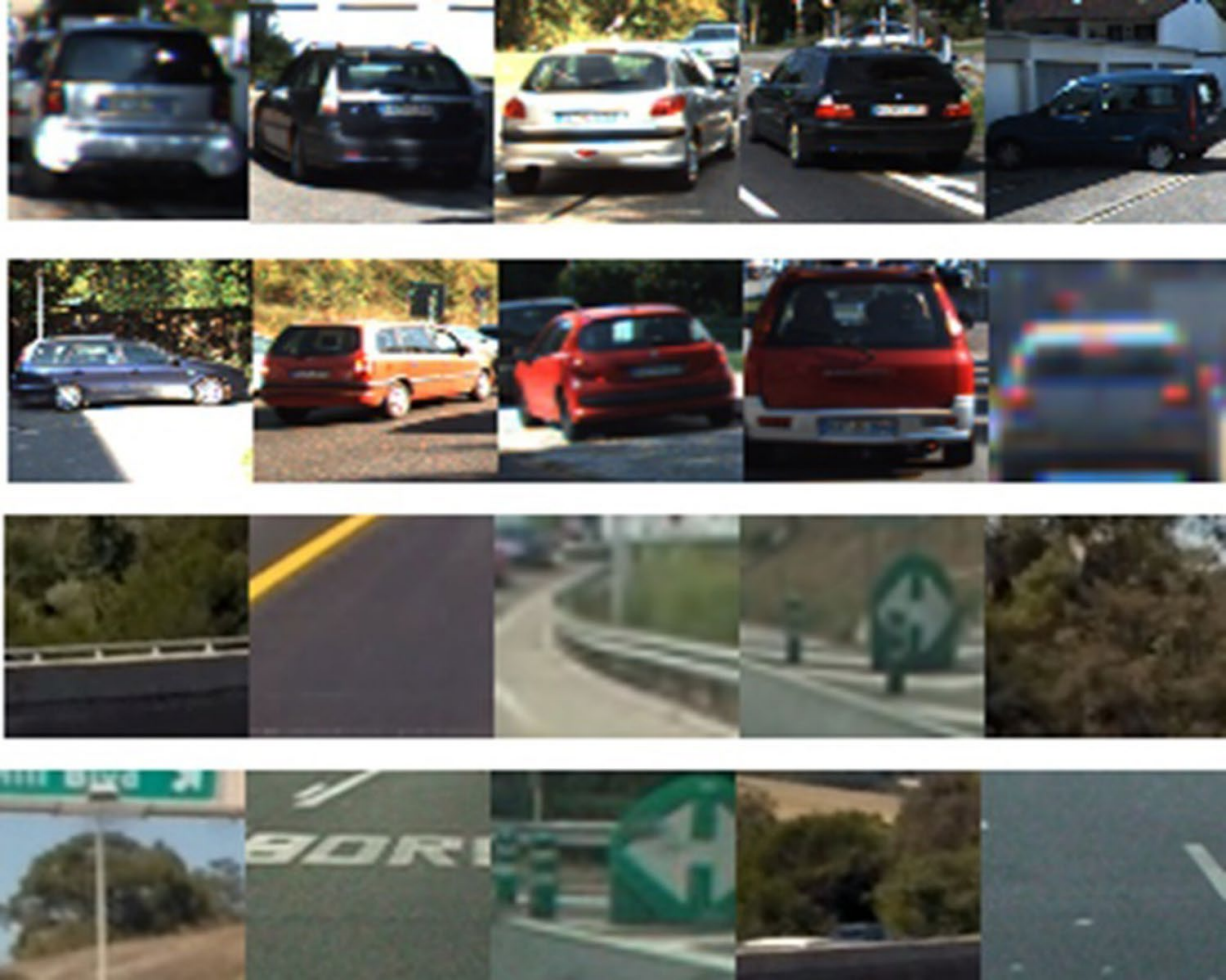
Fragment from data sets

The intelligent car control using vision-based vehicle detection is designed and implemented using CNN. In the first stage, the learning algorithm and image data set are used for the design of CNN based vehicle identification system. The used CNN architecture is shown in Fig. 1. 15000 car images and 5000 non-car images from the GTI dataset [47] were used. Figure 4 depicts fragments from data sets. The images from the dataset have been chosen randomly with the 64 × 64 pixels and fed to the CNN input. All image inputs are formed as 64 × 64×3. The dataset is split into %75 training and %25 testing images. During learning the 64 batch sizes were used for input images. The learning rate was taken as 0.001. The 50 training epochs were used for learning.

**Fig. 5:**
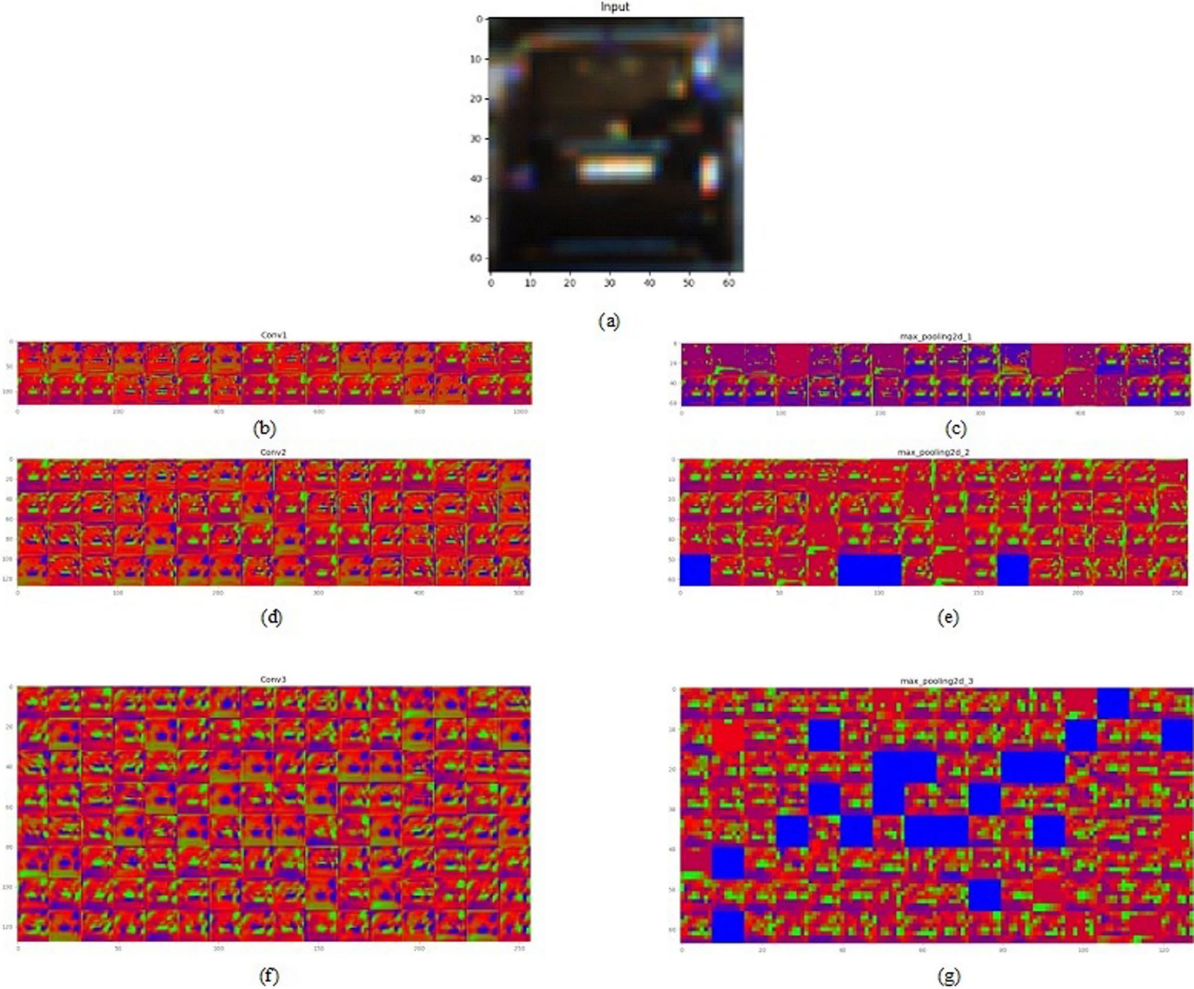
State of outputs of convolution and pooling layers: **a** input of CNN, **b** output of 1-st convolution layer, **c** output of 1-st pooling layer, **d** output of 2-nd convolution layer, **e** output of 2-nd pooling layer, **f** output of 3-rd convolution layer, **g** output of 3-rd pooling layer

The architecture of the designed CNN is presented in Table 1. The first convolutional layer uses 32 filters of size 3 × 3 for each input image. Figure 5 depicts the input of the CNN and the states of the outputs of the convolutional and pooling layers. The outputs of 1-st convolutional layer and pooling layer are given in Fig. 5b, c correspondingly.

**Table 1:** The structure of the CNN model

Type of layers	Output shape	Number of parameters
Input layer		
Input	64 × 64 × 3	0
Hidden layer 1		
Conv1	64 × 64 × 32	896
ReLU	64 × 64 × 32	0
Pool1	32 × 32 × 32	0
Hidden layer 2		
Conv2	32 × 32 × 64	18496
ReLU	32 × 32 × 64	0
Pool2	16 × 16 × 64	0
Hidden layer 3		
Conv3	16 × 16 × 128	73856
ReLU	16 × 16 × 128	0
Pool3	8 × 8 × 128	0
Classification layer		
Flatten	8192	0
Dense1	16	131088
ReLU	16	0
Dense2	64	1088
ReLU	64	0
Dense3	128	8320
ReLU	128	0
Dense4	2	258

Total parameters: 234,002

**Fig.6:**
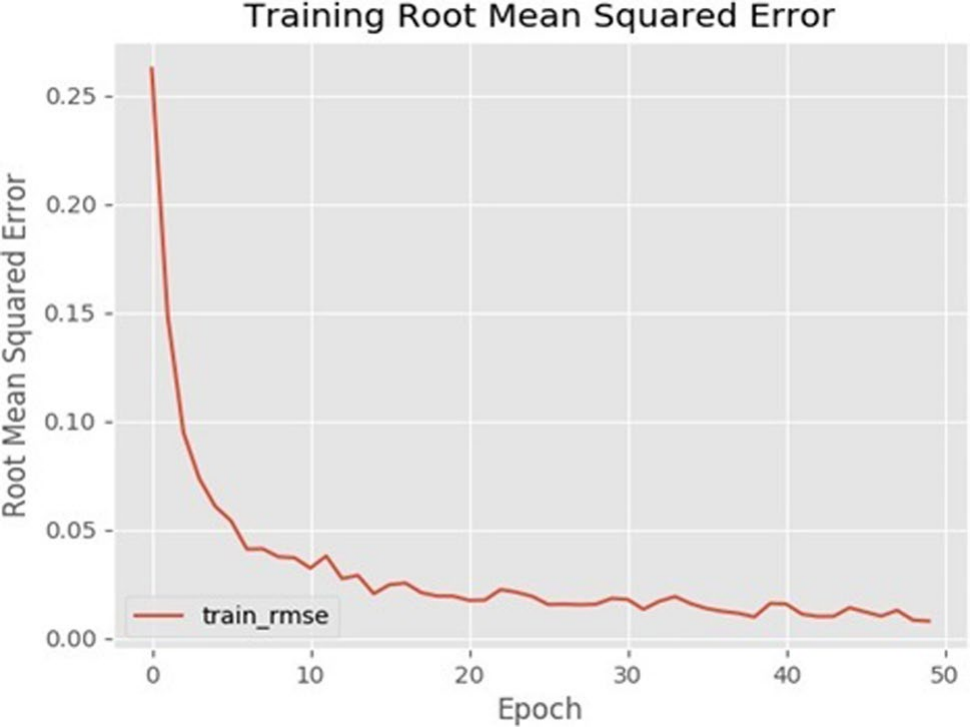
The plot of RMSE values of CNN

In the second layer, 64 convolutional filters of size 3 × 3 and ReLU activation function followed by 2 × 2 max pooling were applied. Figure 5d, e present the output of the 2nd convolutional and pooling layers of CNN, correspondingly. The third layer uses 128 convolutional filters of size 3 × 3 and ReLu activation function followed by the same pooling parameters. Figure 6f, g present the output of the 3rd convolutional and pooling layers, correspondingly.

As shown in Table 1, the fully-connected layer has three dense layers. There are 16 units in the first dense layer, 64 units in the second dense layer and 128- in the next third dense layers. The first layer is used for distributing input signals. ReLU activation is used between layers in order to prevent overfitting. A fully connected layer uses the 1D feature vector as input. Two classes are used in the simulation. These are 1 (presence of the vehicle or obstacle on the road) and 0 (the road is empty). The output layer has two neurons (one for each class) that use the softmax activation function.

After training the designed CNN system is used for the detection of vehicles. The input of CNN is the images of vehicles obtained from a video camera located in the front of the car. The video camera sends 30 images in seconds obtained from the road to the system input. CNN uses the images and determines the presence of the vehicles. Using the CNN the control of car is implemented.

As pointed out above the construction of the vision-based vehicle detection for car driving includes learning and test stages. During learning, in the data augmentation stage, the generator technique was used. The technique includes random rotations, crops, and shifts of the images for getting more precise results by using smaller datasets. Adam optimizer [46] is applied for learning. To estimate the learning and testing performances the root mean squared error has been chosen as a loss function. Figure 6 depicts the RMSE values obtained during training. The training accuracy was obtained as %99.41.

The simulation of the CNN-based vehicle detection system is performed using four different structures described in Table 2. All the models are trained for 50 epochs. For each of the training, validation and testing stages the performance of the system is measured. During simulation, the values of RMSE, Accuracy (ACC), True Positive (TP), False Positive (FP), True Negative (TN), False Negative (FN), Specificity (SPE), Sensitivity (SENS) and Area Under Curve (AUC) are calculated. Table 3 depicts the results for the training, validation and testing stages of the model 1. In the table, the best results are blackened. As shown the performance of model 2 is better than other ones. For this model, the value of accuracy in train, validation and testing stages are obtained as 0.9941, 0.9956 and 0.9981 correspondingly. We test the results using different bath sizes (8, 16,32 and 64). The best results were obtained when the batch size was 64.

**Table 2:** Types of CNN models used in the simulation

Name	Structure	Detail
Model 1	32—> 64—> 128	With dropout layers between convolutional layers
Model 2	32—> 64—> 128	Without dropout layers
Model 3	16—> 64—> 128	With dropout layers between convolutional layers
Model 4	16—> 64—> 128	Without dropout layers

**Table 3:** Performances of the CNN for models 1, 2, 3 and 4

Model	Training			Validation			Testing						
	RMSE	AUC	ACC	RMSE	AUC	ACC	TP	FP	TN	FN	SPE	SENS	ACC
1	0.0125	0.9990	0.9915	**0.0059**	0.9992	**0.9968**	4984	16	4990	10	0.9968	0.9979	0.9974
2	**0.0098**	**0.9995**	**0.9941**	0.0072	**0.9998**	0.9956	4991	9	**4990**	**10**	0.9981	**0.9980**	**0.9981**
3	0.0141	0.9985	0.9913	0.0081	0.9992	0.9952	4989	11	4983	17	0.9977	0.9966	0.9972
4	0.0130	0.9991	0.9919	0.0078	0.9986	0.9956	**4996**	**4**	4976	24	**0.9991**	0.9952	0.9972

Each model is tested using different image resolutions. Table 4 depicts the simulation results of model 2 for image sizes 8 × 8, 16 × 16, 32 × 32 and 64 × 64. In the raining and evaluation stage, the accuracy of the 32 × 32 version was better, but in the testing stage, the result of 64 × 64 was better (0.9981) than others.

**Table 4:** Performances of the CNN (Model 2) using different image’s width and height

WxH	Training			Validation			Testing						
	RMSE	AUC	ACC	RMSE	AUC	ACC	TP	FP	TN	FN	SPE	SENS	ACC
8 × 8	0.0329	0.9977	0.9800	0.0235	0.9976	0.9820	4941	59	4957	43	0.9882	0.9913	0.9898
16 × 16	0.0173	0.9989	0.9894	0.0097	0.9998	0.9936	4991	9	4973	27	0.9981	0.9946	0.9964
32 × 32	**0.0079**	**0.9995**	**0.9952**	**0.0055**	0.9992	**0.9960**	**4993**	**7**	4987	13	**0.9985**	0.9974	0.9980
64 × 64	0.0098	**0.9995**	0.9941	0.0072	**0.9998**	0.9956	4991	9	**4990**	**10**	0.9981	**0.9980**	**0.9981**

The simulation results of the CNN model are compared with the results of the HOG + SVM and FFNN models. The used FFNN was designed using 3 hidden layers respectively with the 512, 256 128 neurons. The comparative results of the used techniques are shown in Table 5. The obtained results indicate that the designed CNN model has better performance than SVM and FFNN models.

**Table 5:** Comparison results of the models

	Accuracy	Sensitivity	Specificity
CR-HOG [45]	94.88	–	–
HOG + SVM	0.912	0.994	0.854
FFNN	0.922	0.886	0.980
CNN	**0.997**	**0.996**	**0.997**

**Fig.7:**
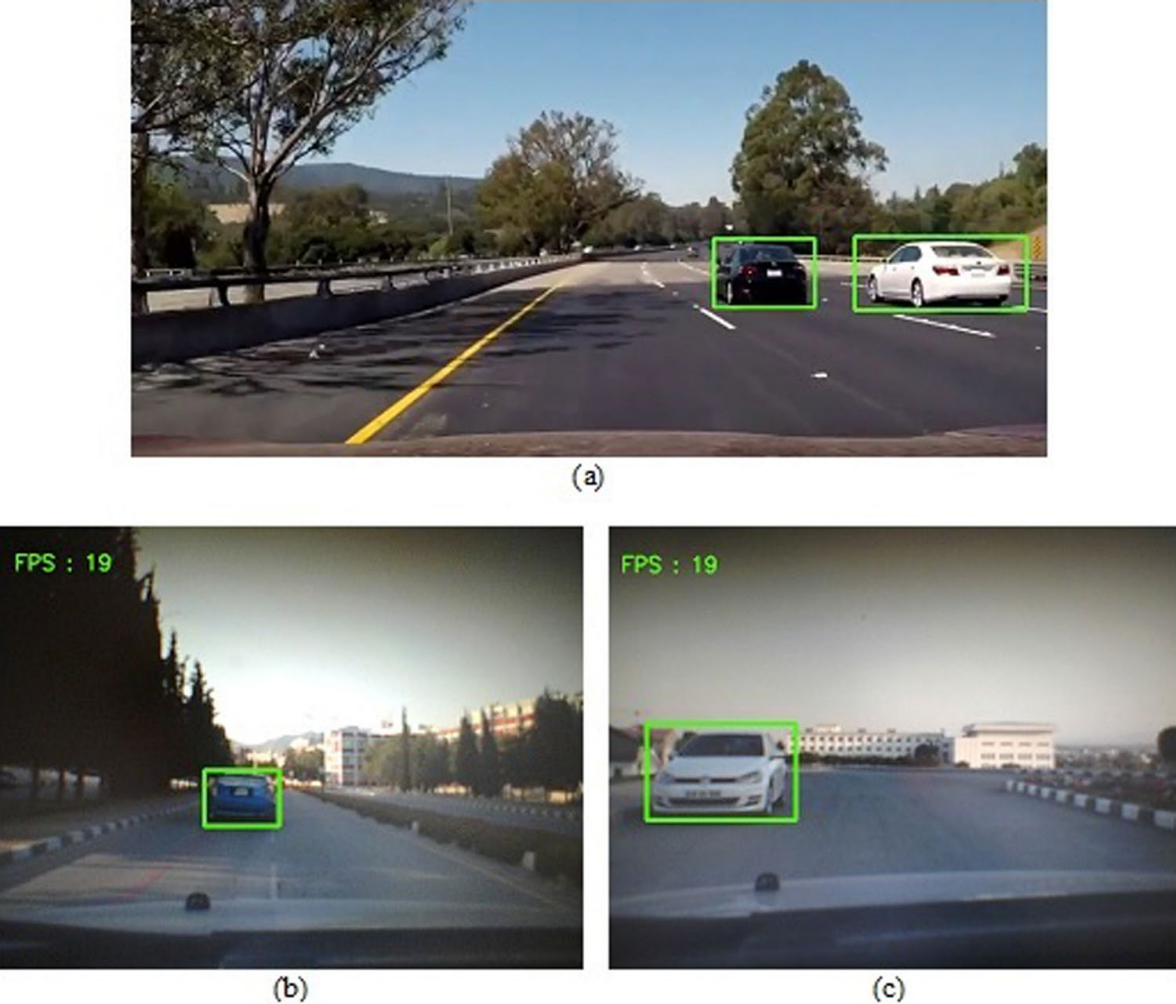
CNN-based vehicle detection for car driving obtained from video cameras

The designed system is tested using traffic videos (Fig. 7). The goal was the real-time detection of vehicles on the road and making a decision on the selection of speed and direction of the controlled car. The images of size 640 × 480 pixels were captured by the video camera which is mounted in front of the car. The sliding window technique is applied to detect vehicles from captured images. We used a 128 × 128 pixel window to slide by 64-pixel step size from left to right in order to detect vehicles. The sky region was dropped and possible car areas are selected by the system. The selected field having a car is resized in 64 × 64 pixels suitable for CNN. These selected areas are used for the recognition (classification) of the cars which is sent to the CNN. Based on input information CNN determined the presence of the car.

## Conclusion

The vision-based vehicle detection system for intelligent car driving is presented. The vehicle detection and recognition system is based on convolutional neural networks. The structure of CNN and its design principles are presented. The advantage of convolutional neural networks is to require low preprocessing steps. The pre-trained model can automatically detect vehicles from the images with simple preprocessing techniques without using image processing such as edge detection, image enhancement, feature extraction and classification techniques. Due to global feature extraction by the convolutional layer, CNN achieved more accurate recognition results for the vehicles from their images. These have been proved by experiments that are based on GTI datasets and achieved more accurate results than other techniques. Experimental results prove that this kind of system is more robust to variations. The designed system can be used in real life and assist the drivers.

## Data Availability

The data that support the findings of this study are openly available in Vehicle Image Database at https://www.gti.ssr.upm.es/data/Vehicle_database or in https://www.kaggle.com/datasets/iamprateek/vehicle-images-gti.
